# Correlation between indoor air pollution and adult respiratory health in Zunyi City in Southwest China: situation in two different seasons

**DOI:** 10.1186/s12889-019-7063-z

**Published:** 2019-06-10

**Authors:** Shixu Li, Jie Xu, Zhigang Jiang, Ya Luo, Yu Yang, Jie Yu

**Affiliations:** School of Public Health, Zunyi Medical University, Zunyi, Guizhou 563000 People’s Republic of China

**Keywords:** Asthma, Asthma-related symptoms, Adult, Indoor, Season

## Abstract

**Background:**

Indoor environmental quality significantly influences the occurrence of asthma attack. Zunyi District has abundant coal reserves and is regarded as one of the cities that are most severely polluted by high levels of particulate matter in China. This study aimed to examine the correlation of indoor exposure with adult respiratory health, as well as the differences in effect between winter and summer.

**Methods:**

A cross-sectional epidemiological study was conducted among 1207 adult residents in Zunyi, Guizhou Province of Southwest China in winter and summer. Data on health variables related to asthma and home environmental factors were collected using a modified European Community Respiratory Health Survey II questionnaire. The following data were obtained: samples of particulate matter 2.5 (PM_2.5_) inside and outside the households under study (*n* = 20); lung function status, including peak expiratory flow rate, forced vital capacity (FVC), forced expiratory volume in 1 s (FEV_1_), and FEV_1_/FVC ratio.

**Result:**

The odds ratio (OR) for asthma-like symptoms and asthma in adults using coal stove for cooking or warming, relative to non-users, was 1.73 (95% CI, 1.11–2.69) in winter vs. 1.30 (95% CI, 0.79–2.14) in summer. Adult residents with exposure to cooking oil fumes were at a considerably higher risk of asthma-like symptoms and asthma [OR = 2.65 (95% CI, 1.25 to 5.61) in winter vs. OR = 7.93 (95% CI, 2.54 to 24.75] in summer] than those without such exposure. The prevalence of asthma-like symptoms and asthma was significantly higher in adults with high kitchen risk scores or high sleeping-area risk scores than in those with low scores in both seasons (*p* < 0.05). The relative kitchen and sleeping area PM_2.5_ concentrations were higher in winter than in summer (*p* < 0.05). Lung function was negatively associated with indoor kitchen and sleeping area relative PM_2.5_ concentration in winter rather than summer (*p* < 0.001). The effect of exposure to indoor risk factors on lung function among the residents was greater in winter than in summer (*p* < 0.001).

**Conclusion:**

Exposure to indoor risk factors, such as aerocontaminants from coal combustion, causes asthma symptoms and reduces pulmonary function. The effect of indoor risk factors on respiratory health among adults with such exposure was greater in winter than in summer.

**Electronic supplementary material:**

The online version of this article (10.1186/s12889-019-7063-z) contains supplementary material, which is available to authorized users.

## Background

Indoor air pollution (IAP) is considered one of the major human health concerns in modern society as people spend approximately 90% of their time indoors, particularly at their own homes. Three billion people worldwide are exposed daily to aerocontaminants of IAP owing to the use of solid fuels such as coal or biomass fuels for combustion. Such use leads to the release of products of incomplete combustion (i.e., particulate matter 2.5 (PM_2.5_)) [1].

An increasing number of studies have indicated that indoor air pollution, as well as lifestyle, contributes to the high prevalence rate of asthma and deterioration of pulmonary function [2–4]. Recent epidemiologic studies showed that asthma-like symptoms and asthma among adult residents in Zunyi have a prevalence of 13.1% in winter [2]. Chronic lung diseases and respiratory tract cancers are strongly associated with pollution from coal burning and other solid fuels [3]. In Europe and North America, studies have demonstrated that even short-term changes in indoor air pollution, other than meteorological conditions, can increase respiratory morbidity in winter or in summer [4, 5]. Several studies have been conducted on indoor risk factor and pulmonary health worldwide [6, 7]. However, comparative studies on the respiratory health effects of indoor air pollution in summer and winter have rarely been reported.

The rapid increase in asthma in recent years cannot be attributed to changes in genetic factor, interventions for the increased prevalence of asthma should be focused on environmental factors. Evidence strongly suggests that exposure to indoor risk factors, including fuel combustion, environmental tobacco smoke, and allergens, can significantly trigger and exacerbate asthma morbidity among adults [8]. Indoor particulate matter affects lung function development, aggravates asthma, and causes other respiratory symptoms [9]. Zunyi has a large coal reserve with high levels of indoor air pollution, The correlation between indoor exposure and adult respiratory health, as well the disparities in effect between winter and summer, prompts interest.

## Methods

### Study design and population

**Fig. 1:**
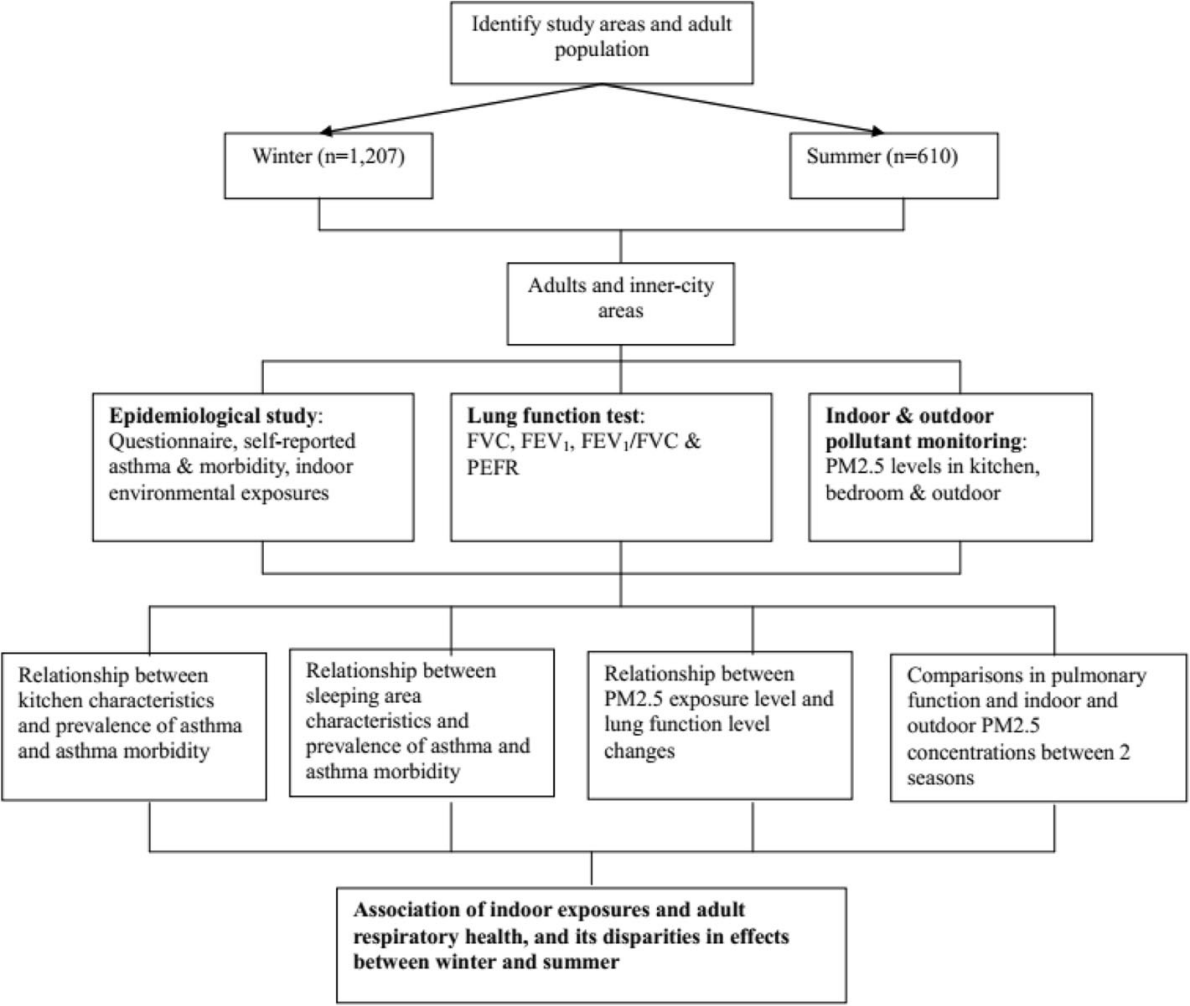
Research flowchart

Adult residents in Zunyi, Guizhou Province in Southwest China were sampled in summer (*n* = 610 from June to August 2012) and in winter (*n* = 1207 from December 2011 to February 2012). Recruitment of the population in this cross-sectional epidemiological study was conducted as described in our previous study [9]. The target group was recruited from 11 downtown areas in Zunyi by multistage cluster sampling. Owing to the relative socioeconomic homogeneity in these areas, one of these areas was randomly sampled in the first stage. Moreover, two of the selected downtown areas, which consisted of 10 residential communities, were randomly sampled in the second stage. The first recruited family in each community was ultimately randomly sampled by residential address. All adults living in the household were asked whether they would agree to participate in the study, and those who agreed were included. Next-door neighbors meeting these inclusion criteria were recruited as well. This procedure was repeated for each house in the selected clusters until the predefined number of residents was reached [10]. A total of 1207 adult residents from 517 households were recruited in winter, and 610 adults from 213 households participated in this study. Among the 1207 residents recruited in winter, 597 could not be traced in summer; meanwhile, the remaining 610 (51.0%) residents participated in the summer survey. The non-traceability of some of the residents was attributed to the transformation of shanty towns, relocation, and refusal, among others. The inclusion criteria for eligible residents were as follows: female or male, age > 18 years, and residence > 3 years in Zunyi City. The exclusion criterion was history of asthma with concomitant diagnoses of chronic obstructive pulmonary disease (chronic bronchitis or emphysema) [2]. A flowchart is presented in Fig. 1.

### Sampling methods

#### Sample size

To determine the sample size of the study, the formulas described by Fleiss, J.L. (1981), which are applicable for cross-sectional studies, were used [11]. With multistage cluster sampling design considered, the design effect on the prevalence of asthma and asthma-related symptoms was estimated to be 2, according to another survey [12]. Ultimately, the total sample size was 1086. The actual survey sample consisted of 1207 adult residents recruited in winter and 610 adult residents recruited in summer.

#### Research tools

The cross-sectional epidemiological study included a questionnaire, spirometric examination, and monitoring of particulate matter (PM_2.5_) pollution. The European Community Respiratory Health Survey II (ECRHS II), a self-administered modified questionnaire, was used to collect data on health variables that typically influence asthma-related symptoms, as well as personal and home environment factors.

The PM_2.5_ concentrations inside (the kitchen and bedroom) and outside the study households while cooking were measured using a real-time digital dust monitor (LD-3 K; Sibata Scientific Technology Inc., Japan) [9]. Air was sampled at a height of 1.2–1.5 m in each household. Within each household, three sites were sampled at 1 m from the cooking stove center. Three sites in the bedroom were randomly sampled at least 3 m away from the kitchen. Three outdoor positions 20 m away from the household were randomly sampled outside the study household as environmental control samples. The average of the three indoor and outdoor samples was determined. Each measurement was maintained for more than one minute, and three readings were utilized to calculate the average relative PM_2.5_ concentration. Monitoring was consistently applied across all households in summer and winter. To determine the relative PM_2.5_ concentration, 11 houses were selected from each residential community by simple random sampling, and 22 houses were selected for measurement in winter. In summer, two houses were not traceable, which was attributed to migration to other areas, leaving only 20 houses for measurement. Indoor and outdoor exposure levels to PM_2.5_ in the 20 houses were measured in winter and summer, and the results were compared.

Data on lung function status, including forced expiratory volume in 1 s (FEV_1_), forced vital capacity (FVC), peak expiratory flow rate (PEFR), and FEV_1_/FVC ratio in the household were determined using a portable electronic FGC-A^+^ spirometer (Anhui Institute of Electronic Science, China) as described in our previous study [9]. The subjects, with feet on the ground and in an upright position, were asked to inhale completely and then exhale forcefully after the meter was put inside their mouths until the lips were sealed around the mouthpiece. This maneuver was demonstrated by an investigator. The maneuvers were only accepted when both FVC and FEV_1_ were within 0.20 L of the best-effort FVC and FEV_1_, back-extrapolated volumes were low (< 5% of the FVC and < 0.15 L), and the final accumulated volume was low in accordance with the practice guideline of the American Thoracic Society. Three expiratory maneuvers were conducted for each subject. The largest FVC of the two curves, the ratio of the largest FEV_1_ to the largest FVC, the largest FEV_1_, and the largest PEFR were analyzed [9].

#### Assignment of scores for potential source of indoor exposure in winter and summer

In this study, 20 potential sources of indoor exposure (19 in summer) concerning kitchen and sleeping area characteristics were identified. Each source of indoor exposure was assigned an exposure score. In summer, the sums of maximum and minimum kitchen risk exposure scores were 22 and 0, respectively, and the sums of sleeping-area risk exposure scores were 19 and 0, respectively. In winter, the sums of maximum and minimum kitchen risk exposure scores were 27 and 0, respectively. Similarly, the sum of the maximum and minimum sleeping-area exposure scores were 23 and 0, respectively. Winter and summer questionnaires, together with instructions for the questionnaire and exposure score, are indicated in Additional files 1, 2, 3.

#### Data analysis

The results were statistically analyzed using SPSS version 20.0. The types of distribution—that is, whether they are normal distributions—were ascertained. Wilcoxon signed-rank test was used for paired comparison between the pulmonary function (FVC, FEV_1_, FEV_1_/FVC, and PEFR) of adults in winter and in summer. Meanwhile, the Mann–Whitney U test was used for comparison between indoor and outdoor relative PM_2.5_ concentrations. Logistic regression was conducted to determine the effects of indoor kitchen, sleeping area, and environmental tobacco smoke (ETS) exposure on the prevalence of asthma-like symptoms and asthma in adults, with other sociodemographic factor variables as controls. Chi-squared tests were conducted to compare the prevalence of asthma symptoms between subjects with high kitchen risk scores and those with low kitchen risk scores. Spearman’s correlation was employed to determine the correlation between PM_2.5_ exposure and pulmonary function in adults in winter. A *P*-value < 0.05 was considered significant.

## Results

### Population characteristics

In this study, 1207 adult residents participated in the survey conducted in winter and 610 participated in the survey conducted in summer. The residents had a mean age of 45.5 y, 51.3% of which were female. Moreover, 4.4% of the total consisted of ethnic minorities, 83% were married, 64% were at least high school-educated, 20% were overweight (BMI ≥ 23 kg/m^2^), 22% experienced asthma-like symptoms and asthma attacks during childhood, 31% reported familial history of asthma-like symptoms and asthma, 80% had high household income (monthly per capita income > US $300), and 20% reported occupational exposure to dust/gas. The characteristics of the 1207 residents are listed in Table 1, as described in our previous study [2] (Table 1).

**Table 1:** Socio-demographic characteristics of the respondents

Socio-demographic characteristics	Subject	
	Number (*n* = 1207)	Percentage (%)
Gender		
Male	588	48.7
Female	619	51.3
Age distribution (years)		
18–39	493	40.8
40–59	485	40.2
≥ 60	229	19.0
Ethnic group		
Han	1154	95.6
Ethnic of minority	53	4.4
Marital status		
Not-married	208	17.2
Married	999	82.8
Education		
Senior high school and above	771	63.9
Below senior high school	436	36.1
BMI (kg/m^2^)		
Underweight (BMI < 18.5 kg/m^2^)	212	17.6
Normal weight (18.5 ≤ BMI < 23 kg/m^2^)	757	62.7
Overweight (BMI ≥ 23 kg/m^2^)	238	19.7
Asthma-like symptoms and asthma in childhood		
Yes	268	22.2
No	939	77.8
Familial history of asthma-like symptoms and asthma		
Yes	375	31.1
No	832	68.9
Monthly household income		
Low household income	247	20.5
High household income	960	79.5
Occupational exposure to dust or gas		
Yes	242	20.0
No	965	80.0

More adults opened their kitchen windows in summer (83.9%) than in winter (74.7%) (*p* < 0.001), with statistically significant difference. A coal stove was used to warm or cook food by 38.8% of the residents in winter vs. 8.1% in summer (*p* < 0.001). Cooking time of 60 min daily was reported by 7.6% of the residents in winter vs. 14.8% in summer (*p* < 0.001). About 75.6% of the adults used a fan or a range hood in their kitchen in winter vs. 3.0% in summer (p < 0.001). Pest-infested kitchen was reported by nearly 1% of adults in winter vs. 5% in summer (*p* < 0.05) (Table 2).

**Table 2:** Comparison between indoor (kitchen, sleeping area, ETS) risk exposures among respondents in winter and summer

Variables	Winter	Summer	χ^2a^	*p* value
	Number of respondents (percentage,%) (n = 1207)	Number of respondents (percentage,%) (*n* = 610)		
Kitchen location				
Separated from other rooms	1158 (95.9)	587 (96.2)	0.089	0.756
Within living rooms or bedrooms	49 (4.1)	23 (3.8)		
Kitchen size				
≥ 4 m^2^	1042 (86.3)	526 (86.2)	0.003	0.095
< 4 m^2^	165 (13.7)	84 (13.8)		
Frequency of opening kitchen windows				
Occasionally or never	45 (3.7)	0 (0)	49.822	< 0.001***
Sometimes	118 (9.8)	20 (3.3)		
Most of the time	142 (11.8)	78 (12.8)		
Always	902 (74.7)	512 (83.9)		
Stove used for cooking or warming				
Clean fuel stove	476 (39.4)	371 (60.8)	188.560	< 0.001***
Fuel mix stove	263 (21.8)	190 (31.1)		
Coal stove	468 (38.8)	49 (8.1)		
Duration of cooking per day				
< 30 min	581 (48.1)	384 (63.0)	90.253	< 0.001***
30–60 min	534 (44.2)	136 (22.3)		
> 60 min	92 (7.6)	90 (14.8)		
Cooking oil fumes				
Never or seldom	819 (67.9)	427 (70.0)	0.866	0.352
Frequently or sometimes	388 (32.1)	183 (30.0)		
Frequency of fan or range hood usage				
Never	65 (5.4)	22 (3.6)	102.680	< 0.001***
Seldom	46 (3.8)	107 (17.5)		
Sometimes	183 (15.2)	97 (15.9)		
Always	913 (75.6)	384 (3.0)		
Kitchen infested with pests				
Never	1071 (88.7)	553 (86.2)	7.730	0.021*
Seldom	122 (10.1)	43 (8.4)		
Sometimes	14 (1.2)	14 (5.4)		
Person(s) sharing in one bedroom				
≥ 3 persons	222 (18.4)	113 (18.5)	0.005	0.945
< 3 persons	985 (81.6)	497 (81.5)		
Carpet				
No	1134 (94.0)	579 (94.9)	0.701	0.402
Yes	73 (6.0)	31 (5.1)		
Carpet use history (years)				
≤ 1 y	1143 (94.7)	584 (95.7)	1.412	0.494
1–5 y	49 (4.1)	18 (3.0)		
> 5 y	15 (1.2)	8 (1.3)		
Mattress material				
Cloth or no mattress	785 (65.0)	503 (82.5)	83.037	<0.001***
Foam or grass/grain husks	251 (20.8)	95 (15.6)		
Feather or hairpiece	171 (14.2)	12 (2.0)		
Mattress use history (years)				
≤ 1 y	509 (42.2)	279 (45.7)	2.156	0.340
1–5 y	582 (48.2)	274 (44.9)		
> 5 y	116 (9.6)	57 (9.3)		
Pillow material stuffed				
Cloth or no pillow	954 (79.1)	535 (87.7)	45.642	<0.001***
Grass or foam	127 (10.5)	65 (10.7)		
Feather	126 (10.4)	10 (1.6)		
Keep pets				
No	937 (68.9)	487 (79.8)	106.246	<0.001***
Yes	270 (31.1)	123 (20.2)		
Pet allowed in bedroom				
No	1135 (94.0)	568 (93.1)	0.583	0.445
Yes	72 (6.0)	42 (6.9)		
Water damage				
No	1112 (92.1)	575 (94.3)	2.775	0.096
Yes	95 (7.9)	35 (5.7)		
Musty air in bedroom				
No	1108 (91.8)	586 (96.1)	11.694	0.001**
Yes	99 (8.2)	24 (3.9)		
Mold in bedroom				
No	1122 (93.0)	593 (97.2)	13.848	<0.001***
Yes	85 (7.0)	17 (2.8)		
New furniture				
No	1119 (92.7)	593 (97.2)	15.097	<0.001***
Yes	88 (7.3)	17 (2.8)		
Decoration and fitment				
No	1147 (95.0)	599 (98.2)	10.829	0.001**
Yes	60 (5.0)	11 (1.8)		
Smoking status				
Non-smokers	688 (57.0)	365 (59.8)	1.803	0.406
Ex-smokers	167 (13.8)	85 (13.9)		
Current smokers	352 (29.2)	160 (26.2)		
Exposure to second-hand smoke, ETS				
Yes	222 (18.4)	518 (84.4)	742.80	< 0.001***
No	985 (81.6)	92 (15.6)		

Values are number (%). ^a^ Chi-squared test, α = 0.05, *significant at *p* < 0.05, **significant at *p* < 0.01, *** significant at *p* < 0.001

With regard to sleeping-area risk factors, a feather or hairpiece mattress in winter was used by 14.2% of the adults in winter vs. 2.0% in summer (*p* < 0.001). About 31.1% of the adults had indoor pets in winter vs. 20.2% in summer (p < 0.001). Mold growth was reported by 7.0% of the adults in winter vs. 2.8% in summer (*p* < 0.001). More than 7% of the adults had new furniture in winter vs. 2.8% in summer. Domestic decorations and fitment were used by 5.0% of the adults in winter vs. 1.8% in summer (*p* < 0.001). Nearly 18% of the residents reported exposure to second-hand smoke in winter vs. 84% in summer (*p* < 0.001) (Table 2).

### Comparison of the prevalence of asthma-like symptoms and asthma as well as outcomes of logistic regression analysis between winter and summer

Table 3 presents the estimated effects of kitchen location, smoking status, cooking oil fumes, pets, stove used for cooking or warming, and second-hand smoke, mattress use history, and mold in bedroom in winter and/or summer, in addition to selected socioeconomic and demographic variables, on the prevalence of such symptoms suffered by the adult residents.

**Table 3:** Comparison of the prevalence of asthma-like symptoms and asthma and outcome of logistic regression analysis in Zunyi in winter vs. summer

Risk factors	Winter				Summer			
	Prevalence of asthma-like symptoms and asthma							
	B	Standard Error	OR (95%CI)	*P* value	B	Standard Error	OR (95%CI)	*p* value
Constant	−13.655	1.785	0.000	<0.001***	−8.255	1.511	0.000	<0.001***
Age, years	0.029	0.014	1.030 (1.003,1.058)	0.030*	0.031	0.013	1.032 (1.006,1.05)	0.016*
BMI	0.548	0.226	1.730 (1.111,2.693)	0.015*	0.262	0.253	1.299 (0.791,2.135)	0.301
Asthma and asthma-related symptoms in childhood	2.505	0.391	12.239 (5.690,26.329)	<0.001***	1.928	0.424	6.877 (2.994,15.767)	<0.001***
Familial history of asthma-like symptoms and asthma	0.897	0.361	2.452 (1.209,4.971)	0.013*	0.660	0.410	1.934 (0.866,4.319)	0.107
Kitchen location	0.714	0.923	2.042 (0.334,12.478)	0.439	2.176	0.812	8.807 (1.794,43.241)	0.007**
Stove used for cooking or warming	0.608	0.217	1.836 (1.200,2.810)	0.005**	0.841	0.276	2.318 (1.349,3.982)	0.002**
Cooking oil fumes	0.973	0.384	2.646 (1.247,5.613)	0.011*	2.070	0.581	7.926 (2.538,24.753)	<0.001***
Keep pets	1.087	0.403	2.966 (1.347,6.527)	0.007**	−0.858	0.654	0.424 (0.118,1.526)	0.189
Smoking status	0.984	0.240	2.675 (1.670,4.284)	<0.001***	0.436	0.236	1.546 (0.973,2.457)	0.065*
Second-hand smoke	1.377	0.401	3.963 (1.807,8.691)	<0.001***	1.082	0.483	2.950 (1.144,7.607)	0.025*
Mattress use history	–	–	–	–	−1.270	0.354	0.281 (0.140,0.562)	<0.001***
Mold in bedroom	–	–	–	–	2.041	0.713	7.696 (1.903,31.127)	0.004**

Logistic regression, α = 0.05, *significant at *p* < 0.05, **significant at *p* < 0.01, *** significant at *p* < 0.001

Differences in factors causing asthma-like symptoms and asthma in Zunyi were observed between summer and winter. After adjustments for host factors, such as gender and educational level, an increase of one year in age was found to have a 2.9% increase in asthma-like symptoms and asthma [95% confidence interval (CI), 1.00–1.06] in winter vs. 3.1% (95% CI, 1.01–1.05) such increase in summer. The odds ratio (OR) for asthma-like symptoms and asthma in adults with BMI of at least 23.0 kg/m^2^ relative to BMI < 18.5 kg/m^2^ was 1.73 (95% CI, 1.11–2.69) in winter vs. 1.30 (95% CI, 0.79–2.14) in summer. Adult residents who experienced asthma-like symptoms and asthma in childhood were significantly associated with those who experienced asthma-like symptoms and asthma in adulthood, with OR of 12.24 (95% CI, 5.69–26.33) in winter vs. 6.88 (95% CI, 2.99–15.77) in summer. Subjects with a family history of asthma also showed significantly higher prevalence of asthma-like symptoms and asthma compared with subjects without such history [OR = 2.45 (95% CI, 1.21–4.97) in winter vs. OR = 1.93 (95% CI, 0.87–4.32) in summer]. Among the home environment factors, the coal stove used for cooking or warming and the prevalence of adult asthma-like symptoms and asthma exhibited a statistically significant association [OR = 1.83 (95% CI, 1.20–2.81) in winter vs. OR = 2.3 (95% CI, 1.35–3.98) in summer]. Adult residents with exposure to cooking oil fumes were at a considerably higher risk of suffering from such symptoms [OR = 2.65 (95% CI, 1.25–5.61) in winter vs. OR = 7.93 (95% CI, 2.54–24.75) in summer], compared with those without such exposure. Compared with the controls, the adult residents with pets were 2.97 times more likely to develop such symptoms in winter (OR = 2.97; 95% CI, 1.347–6.527); meanwhile, having pets can be a protective factor for asthma-like symptoms (OR = 0.42; 95% CI, 0.12–1.53) in summer. The adjusted ORs for experiencing asthma-like symptoms and asthma were nearly 3 times higher in winter vs. 1.5 times higher in summer among the subjects who were then smokers, compared with those who had never smoked (OR = 2.68; 95% CI, 1.670–4.284 vs. OR = 1.55; 95% CI, 0.97–2.46). The odds of suffering from asthma-like symptoms and asthma were about 4 times higher in winter vs. 3 times higher in summer among the adult residents with exposure to second-hand smoke than those without such exposure (OR = 3.96; 95% CI, 1.81–8.70 vs. OR = 2.95; 95% CI, 1.14–7.61). In addition, in summer rather than winter, the risk of asthma attack was about 8 times (OR = 7.70, 95% CI, 1.90–31.13) higher among subjects with exposure to molds than those without such exposure. With other controlled variables, history of mattress use > 5 years also seemed to be a protective factor (OR = 0.28; 95% CI, 0.14–0.56) for asthma and asthma morbidity (Table 3).

### Effects of environment and personal risk factors on asthma-like symptoms and asthma in winter and summer

In the winter survey, 158 of the 1207 adult residents reported experiencing asthma-like symptoms and asthma, whereas 1049 adults reported no such experience. In reports without asthma and asthma related symptoms, the median (25th and 75th percentiles) kitchen risk score and sleeping-area risk score were 6.0 (4.0–7.0) and 2.0 (1.0–4.0), respectively. The median (25th and 75th percentiles) kitchen risk scores among the subjects with such symptoms and those without such symptoms were 6.0 (5.0–8.0) and 6.0 (4.0–7.0), respectively. Significant difference was indicated between those with and without such symptoms (*p* < 0.001). The median (25th and 75th percentiles) scores for the sleeping area risk factor among the subjects with and without such symptoms were 3.0 (1.0–5.0) and 2.0 (1.0–4.0), respectively, The difference between those with and without such symptoms was statistically significant (p < 0.001) (Table 4).

**Table 4:** Comparison of the median kitchen risk score and sleeping-area risk score among subjects with asthma-like symptoms and asthma and those without such symptoms in winter and summer

Asthma-like symptoms and asthma	Winter (n = 1207)		Summer (n = 610)	
	Median score for kitchen risk factor (25th, 75th percentiles)	Median score for sleeping- area risk factor (25th, 75th percentiles)	Median score for kitchen risk factors (25th,75th percentiles)	Median score for sleeping area risk factors (25th,75th percentiles)
Subjects with asthma-like symptoms and asthma	6.0 (5.0–8.0)	3.0 (1.0–5.0)	3.5 (2.0–7.0)	2.0 (0.75–4.25)
Subjects without asthma-like symptoms and asthma (*n* = 1049)	6.0 (4.0–7.0)	2.0 (1.0–4.0)	2.0 (1.0–5.0)	2.0 (1.0–3.0)
Z value	−4.481^a^	−4.007^a^	−3.06^a^	−1.46^a^
*p* value	< 0.001***	< 0.001***	0.002**	0.144

^a^Nonparametric test (Mann–Whitney U test), α = 0.05; *significant at *p* < 0.05, **significant at *p* < 0.01, *** significant at *p* < 0.001

Among the 610 residents surveyed in summer, 46 reported having experienced asthma-like symptoms and asthma, whereas 564 adults reported no such experience. For studies in which asthma and asthma related symptoms were reported, the median (25th and 75th percentiles) kitchen risk score and sleeping-area risk score were 2.0 (1.0–5.0) and 2.0 (1.0–3.0), respectively. The median (25th and 75th percentiles) kitchen risk scores among the subjects with such symptoms and those without such symptoms were 3.5 (2.0–7.0) and 2.0 (1.0–5.0), respectively, Significant difference was indicated between those with and without such symptoms (*p* < 0.01). The median (25th and 75th percentiles) scores for the sleeping area risk factor in the two groups were 2.0 (0.75–4.25) and 2.0 (1.0–3.0), respectively, but no significant difference was observed between the two groups (*p* > 0.05) (Table 4).

The median (25th and 75th percentiles) score for the kitchen risk factor was 6.0 (4.0–7.0) among 1207 adults in the winter survey and 2.0 (1.0–5.0) in the summer survey among 610 adults; consequently, the subjects with a kitchen risk score of 6 in winter or 2 and above in summer were categorized into subjects with a high kitchen risk score. Conversely, subjects with kitchen risk scores of 6 and below in winter or 2 and below in summer were classified into subjects with low kitchen risk scores. In the winter survey, 77 adults with high kitchen risk scores experienced asthma-like symptoms and asthma, whereas 81 adults with low kitchen risk scores reported experiencing such symptoms, Significant difference in the prevalence of such symptoms was found between those with high kitchen risk scores and those with low kitchen risk scores (*p* < 0.01). In the summer survey, 30 adults with high kitchen risk scores and 16 adults with low kitchen risk scores experienced such symptoms, respectively. Significant difference in the prevalence of such symptoms was indicated between those with high kitchen risk scores and those with low kitchen risk scores (*p* < 0.05) (Table 5).

**Table 5:** Differences in the prevalence of asthma-like symptoms and asthma between the subjects with high and low kitchen risk scores in winter and summer

Kitchen risk factors	Winter		Summer	
	Asthma-like symptoms and asthma			
	Yes (n)	No (n)	Yes (n)	No (n)
Subjects with high kitchen risk score	77	384	30	267
Subjects with low kitchen risk score	81	665	16	297
Pearson Chi-square	8.556^a^		5.441^a^	
*p* value	0.002**		0.014*	

^a^Chi-square test, α = 0.05; *significant at *p* < 0.05, **significant at *p* < 0.01

Analogously, the median (25th and 75th percentiles) score for the sleeping-area risk factors was 2.0 (1.0–4.0) among the 1207 adults in the winter survey and 2.0 (1.0–3.0) among the 610 adults in the summer survey. Accordingly, the subjects with sleeping-area risk scores of 2 and above in winter or 2 and above in summer were categorized into subjects with high sleeping-area risk scores. By contrast, the subjects with sleeping-area risk scores of 2 and below in winter or 2 and below in summer were categorized into subjects with low sleeping-area risk scores. A total of 92 adults with high sleeping-area risk scores and 66 adults with low sleeping-area risk scores reported having experienced asthma-like symptoms and asthma in the winter survey. Significant difference was observed between those with high or low sleeping-area risk scores (*p* < 0.01). Asthma-like symptoms and asthma were reported in 16 adults with high sleeping-area risk scores and 30 adults with low sleeping-area risk scores in the summer survey; however, no significant difference was found between the two aforementioned groups (*p* > 0.05) (Table 6).

**Table 6:** Difference in the prevalence of asthma-like symptoms and asthma between subjects with high and low sleeping-area risk scores in winter and summer

Sleeping area risk factors	Winter		Summer	
	Asthma-like symptoms and asthma			
	Yes (n)	No (n)	Yes (n)	No (n)
Subjects with high sleeping-area risk scores	92	480	16	147
Subjects with low sleeping-area-risk scores	66	569	30	417
Pearson Chi-square	8.564^a^		1.651^a^	
*p* value	0.002**		0.134	

^a^Chi-square test, α = 0.05; *significant at *p* < 0.05, **significant at *p* < 0.01

### Comparison of indoor and outdoor relative PM_2.5_ concentrations in winter and summer

**Fig. 2:**
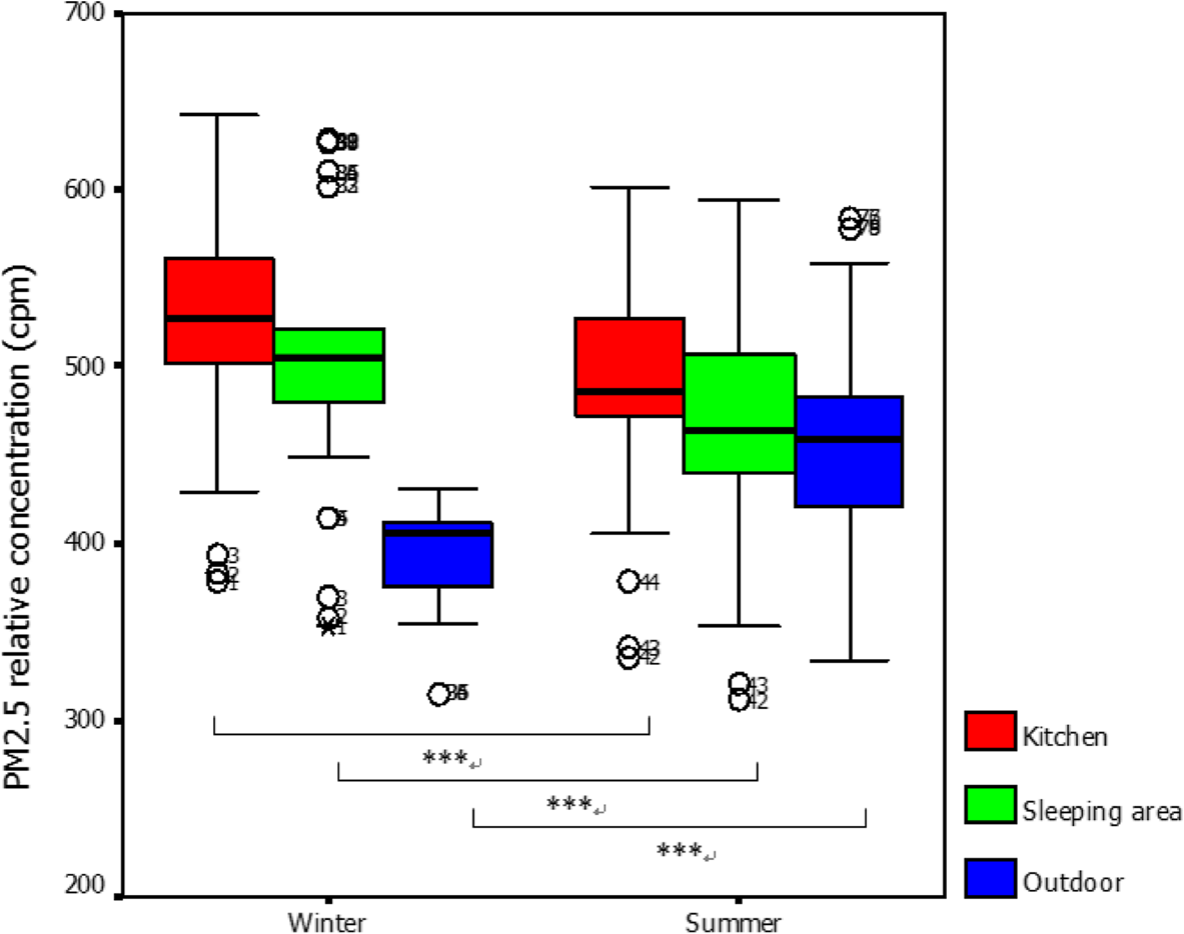
Comparison of relative PM_2.5_ concentrations among 20 selected houses in winter and summer. Mann–Whitney U test, ^***^ significant at *p* < 0.001

Figure 2 shows that the relative PM_2.5_ concentrations (cpm) in the kitchen (z = − 5.583, *p* < 0.001) and sleeping area (z = − 5.587, p < 0.001) in winter were significantly higher than those in summer for all 20 houses. However, the outdoor relative PM_2.5_ concentration (z = − 5.420, *p* < 0.001) in summer was significantly higher than that in winter.

### Comparison of the effects of indoor environmental risk factors on variations in pulmonary function parameters (FVC, FEV_1_, FEV_1_/FVC, and PEFR)

Table 7 compares the pulmonary function in summer with that in winter among 46 residents who reported experiencing asthma-like symptoms and asthma in summer. Wilcoxon signed-rank tests were utilized to determine whether significant difference in changes in pulmonary function (FVC, FEV_1_, FEV_1_/FVC, and PEFR) was present between summer and winter. The FVC, FEV_1_, and PEFR of the subjects in winter were lower than those in summer (*p* < 0.001). The subjects exhibited a significant decrease in FEV_1_/FVC in summer relative to that in winter (*p* < 0.001). Significant differences in the four parameters was observed between summer and winter (*p* < 0.001).

**Table 7:** Paired comparison analysis of pulmonary function (FVC, FEV_1_, FEV_1_/FVC, and PEFR) in 46 adults in winter and summer

Pulmonary function parameters	Asthma-like symptoms and asthma		Z	*p* value
	Summer (*n* = 46)	Winter (n = 46)		
FVC in litres (L)	3.5 (2.7–4.1)	2.9 (2.1–3.7)	−5.91^a^	< 0.001***
FEV_1_ in litres (L)	3.3 (2.4–3.8)	2.7 (1.8–3.5)	−5.91^a^	< 0.001***
FEV_1_/FVC in percentage	91.7 (87.7–94.0)	91.9 (82.0–94.6)	−2.91^a^	< 0.001***
PEFR in litres/min	382.8 (324.9–420.7)	347.9 (303.8–404.4)	−5.89^a^	< 0.001***

^a^Nonparametric test (Wilcoxon signed-rank test); *significant at *p* < 0.05; *significant at *p* < 0.05, **significant at *p* < 0.01, *** significant at *p* < 0.001

### Relationship between indoor and outdoor relative PM_2.5_ concentrations and pulmonary function (FVC, FEV_1_, FEV_1_/FVC, and PEFR) in winter and summer

**Fig. 3:**
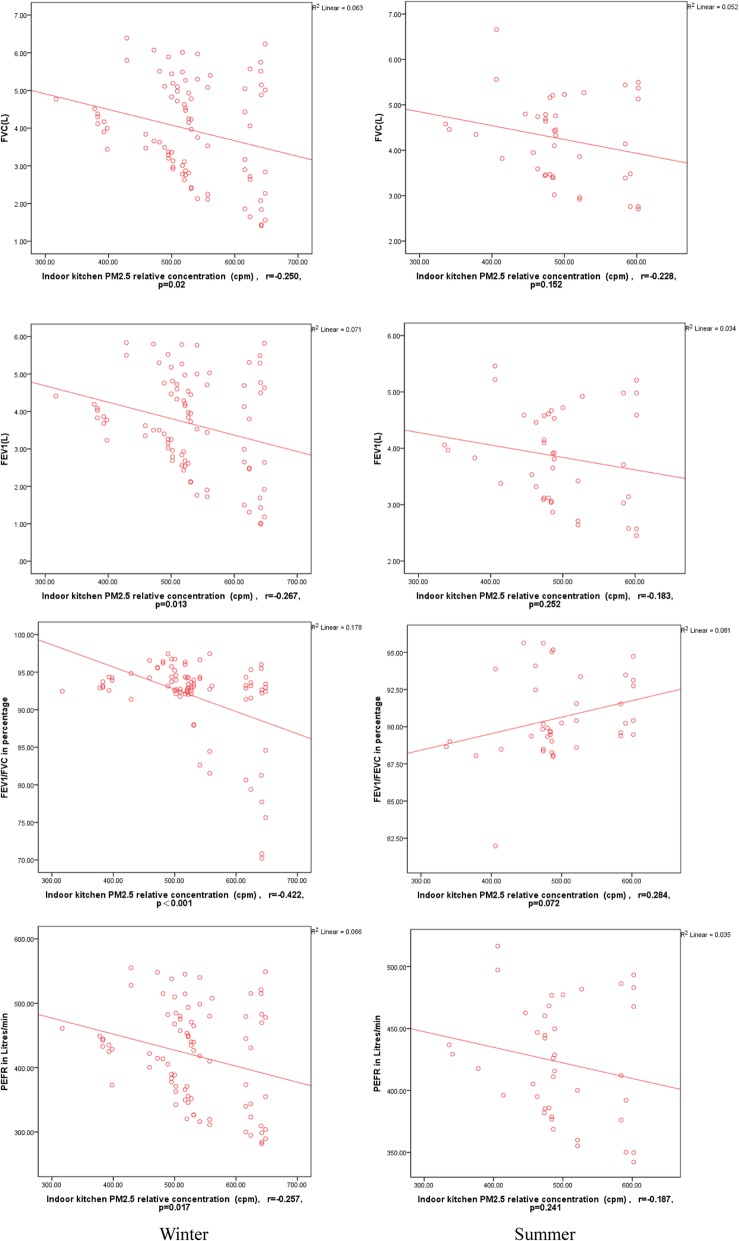
Comparison of pulmonary function (FVC, FEV_1_, FEV_1_/FVC, and PEFR) in the adults and correlation of pulmonary function with kitchen PM_2.5_ exposure in winter and summer. Spearman correlation, r: Correlation coefficient

**Fig. 4:**
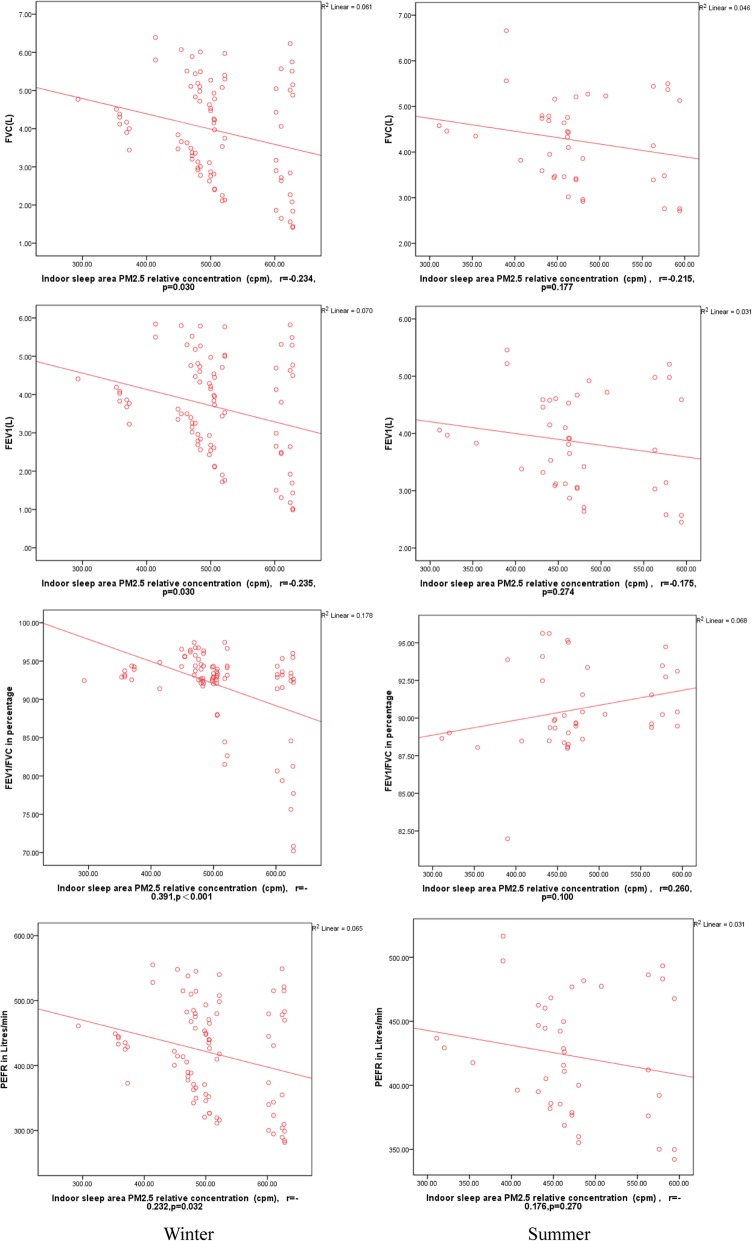
Comparison of pulmonary function (FVC, FEV_1_, FEV_1_/FVC, and PEFR) in the adults and correlation of pulmonary function with sleeping area PM_2.5_ exposure in winter and summer. Spearman correlation, r: Correlation coefficient

Figures 3 and 4 show that a significant negative correlation exists between the pulmonary function test parameters of 86 adult residents and the relative PM_2.5_ concentrations of the indoor kitchen in the winter survey; however, no significant correlation was found in the summer survey [13] (FVC: r = − 0.250, *p* = 0.020 vs. r = − 0.228, *p* = 0.152; FEV_1_: r = − 0.267, *p* = 0.013; FEV_1_/FVC: r = − 0.422, *p* < 0.001 vs. r = 0.284, *p* = 0.072; PEFR r = − 0.257, *p* = 0.017 vs. r = − 0.187, *p* = 0.241). The pulmonary function test parameters and the relative PM_2.5_ concentrations of the indoor sleeping area exhibited a significant negative correlation in the winter survey; however, no significant correlation was observed in the summer survey (FVC: r = − 0.234, *p* = 0.030 vs. r = − 0.215, *p* = 0.177; FEV_1_: r = − 0.235, p = 0.030 vs. r = − 0.175, *p* = 0.274; FEV_1_/FVC: r = − 0.391, p < 0.001 vs. r = 0.260, *p* = 0.100; PEFR r = − 0.232, *p* = 0.032 vs. r = − 0.176, *p* = 0.270) (Fig. 3). In addition, no significant differences in FVC, FEV_1_, FEV_1_/FVC, and PEFR were observed between the pulmonary function test parameters and the relative outdoor kitchen PM_2.5_ concentrations in winter and summer (*p* > 0.05).

## Discussion

The prevalence of asthma-like symptoms and asthma has markedly increased over the last decade in China and Western industrial countries. Indoor environmental quality significantly affects the occurrence of asthma attacks. In this study, exposure to indoor risk factors (e.g., stove used for cooking or warming, cooking oil fumes, and smoking status) was associated with the increased risks of asthma-like symptoms and asthma among adult residents, particularly in winter. The PM_2.5_ levels in the kitchen and the sleeping area were higher in winter than in summer. A negative relationship between lung function and the relative PM_2.5_ concentrations in the indoor kitchen and the sleeping area was also observed in winter rather than summer. The effect of exposure to indoor risk factors on lung function was greater in winter than in summer.

We previously reported that among the various risk factors, asthma in childhood, kitchen in the living room or bedroom, mixed fuel stove, cooking oil fumes, second-hand smoke, mold growth, and home furnishings were associated with increased risks of adult asthma-like symptoms and asthma [2]. Studies have found a similar association between specific indoor environmental exposure and exacerbation of adult asthma [3, 14]. For the first time, potential sources of exposure to indoor air pollutants were quantified in detail and assigned a score for each exposure risk factor to evaluate the relationship between different degrees of exposure to indoor (i.e., in the kitchen and the bedroom) environmental contaminants and asthma morbidity in this study. Our results indicate that both the kitchen risk score and the sleeping-area risk score were significantly higher in adults with asthma morbidity than in those without, particularly in winter. Moreover, the prevalence of asthma-like symptoms and asthma was significantly greater in adults with high kitchen risk scores or high sleeping-area risk scores than in those with low scores in both seasons. These findings suggest that exposure to indoor risk factors, such as aerocontaminants from coal combustion, leads to asthma symptoms and exacerbations. Although an association between exposure to indoor pollutants and childhood asthma has been reported in the last two decades, few studies have focused on adult population.

Residents in underdeveloped areas in China still use stoves for cooking and warming, increasing coal consumption. Fu et al. (2016) conducted a cross-sectional survey by stratified random sampling in 7 cities in China. Coal cooking was found to be an independent determinant of indoor environment for asthma (OR = 2.65) [15]. Kim et al. (2013) showed that coal cooking adversely affects indoor air quality [16]. The results of these two studies were consistent with our study, which found that the coal stove used for cooking or warming was significantly correlated to the prevalence of adult asthma and asthma morbidity in both seasons.

The relationship between indoor air pollution and poor pulmonary function has been demonstrated in numerous studies. In their cross-sectional study in the United States, Stephanie et al. found no significant associations between IAP exposure and pulmonary function in adults [15]. Several studies indicated a positive relationship between indoor environmental exposure and respiratory health. A randomized exposure study of pollution and respiratory effects in the United Kingdom showed an association between exposure to household air pollution from wood combustion and low level of lung function in nonsmoking women [16]. However, data relating indoor PM_2.5_ concentrations to lung function outcomes are limited. The results of our study are consistent with the findings by Yulia [17], that a significant negative correlation exists between pulmonary function and indoor relative PM_2.5_ concentration rather than outdoor relative PM_2.5_ concentration; however, correlation coefficients between − 0.20 and − 0.40 were considered low. An association between exposure to PM_2.5_ from indoor coal combustion and decreased lung function in adults has not been determined.

We found that the relative PM_2.5_ concentrations in the kitchen and the bedroom were higher in winter than in summer. The FVC, FEV_1_, and PEFR were lower in winter than in summer. Coal is the major domestic fuel for cooking and baking and warming households in most Zunyi households, particularly in winter. In winter, combustion of coal and natural gas in poorly ventilated homes exposes children and adults to high levels of PM, sulfur oxides (SO_2_), and other air pollutants in Zunyi. In summer, many households using coal experience CO levels several times the national indoor air quality (IAQ) standard of 10 mg/m^3^ (equivalent to 9 ppm) [18], and in winter, the situation worsens, particularly in households using coal stove. Moreover, risks to respiratory health for many people may be increased because of exposure to excessively high indoor pollutants from poorly ventilated household stoves. The longer a household heats in winter, the more likely its members are to show impaired lung function. Regardless of the type of fuel used, the concentrations of both PM pollutants and SO_2_ were highest in winter when fuel consumption was greatest; meanwhile, the concentrations were lowest in summer when heating requirements were lower.

The current study has a number of limitations. Personal PM_2.5_ monitoring of cooks and noncooks spending most of their time at home to assess individual continuous exposures was not conducted. PM_2.5_ monitoring at home in both seasons was relatively short and should be performed during the entire winter and in summer. The cross-sectional design might find a weak association between risk factor exposure and respiratory health because of confounding from individual risk factors.

## Conclusion

Exposure to indoor risk factors, such as aerocontaminants from coal combustion, has been hypothesized to cause asthma symptoms, as well as exacerbations, and decrease pulmonary function. The effect of exposure to indoor risk factors on respiratory health among adults was greater in winter than in summer.

## Additional files

Additional file 1: Questionnaire in winter. (DOC 118 kb)

Additional file 2: Questionnaire in summer. (DOC 106 kb)

Additional file 3: Instructions for the questionnaire and exposure score. (DOCX 32 kb)

## Data Availability

All data and materials related to the study can be obtained through contacting the correspondent author at Xujie360@hotmail.com.

## Abbreviations

CI: Confidence interval
ECRHS II: European Community Respiratory Health Survey II
ETS: Environmental tobacco smoke
FEV_1_: Forced expiratory volume in 1 s
FVC: Forced vital capacity
IAP: Indoor air pollution
IAQ: Indoor air quality
OR: Odds ratio
PEFR: Peak expiratory flow rate
PM_2.5_: Particulate matter 2.5
