# Correction to: Correlation between indoor air pollution and adult respiratory health in Zunyi City in Southwest China: situation in two different seasons

**DOI:** 10.1186/s12889-019-7285-0

**Published:** 2019-08-05

**Authors:** Shixu Li, Jie Xu, Zhigang Jiang, Ya Luo, Yu Yang, Jie Yu

**Affiliations:** School of Public Health, Zunyi Medical University, Zunyi, Guizhou 563000 People’s Republic of China

**Correction to: BMC Public Health (2019) 19:723**

**https://doi.org/10.1186/s12889-019-7063-z**

It has been highlighted that the original article [1] contained some errors in the Result section of the Abstract. The incorrect and correct statement is shown in the Correction article.

**Incorrect**

The odds ratio (OR) for asthma-like symptoms and asthma in adults using coal stove for cooking or warming, relative to non-users, was 1.73 (95% CI, 1.11–2.69) in winter vs. 1.30 (95% CI, 0.79–2.14) in summer.

**Correct**

The odds ratio (OR) for asthma-like symptoms and asthma in adults using coal stove for cooking or warming, relative to non-users, was 1.84 (95% CI, 1.20–2.81) in winter vs. 2.32 (95% CI, 1.35–3.98) in summer.
