# Supplementary material for: Correlation between indoor air pollution and adult respiratory health in Zunyi City in Southwest China: situation in two different seasons
Source: BMC Public Health. 2019 Jun 10;19:723. doi: 10.1186/s12889-019-7063-z (PMC6558730; doi:10.1186/s12889-019-7063-z)
Supplement: Supplementary file 1 — Questionnaire in winter. (DOC 118 kb) [file 12889_2019_7063_MOESM1_ESM.doc]

Additional file 1:

**AsthmA, asthma-related symptoms questionnaire IN WINTER (English version)**

**AsthmA and asthma-related symptoms questionnaire**

NAME: ________________________________________________________________

(Last) (First) (Middle Initial)

Residential ADDRESS: _______________________________________________________

(Street or Route No) (Zip Code)

TELEPHONE NUMBER: _______________________________________________________

DATE OF QUESTIONNAIRE COMPLETED: [ ][ ] / [ ][ ] / [ ][ ][ ][ ]

Date Month Year

HOW OLD ARE YOU? _____________years old

Occupation: ________________________________

| Sex: |  | Male ___ |
| --- | --- | --- |
|  |  | Female ___ |
|  |  |  |
| Height: |  | ______(cm) |
| Weight: |  | ______(kg) |
|  |  |  |
| What is your marital status? |  | Single___ |
|  |  | Married___ |
|  |  | Widowed___ |
|  |  | Separated/Divorced___ |
|  |  |  |
| Ethnic group: |  | Han___ |
|  |  | Miao___ |
|  |  | Bouyei ___ |
|  |  | Others. Please state ______ |
|  |  |  |
| Education status: |  | Primary school ___ |
|  |  | Secondary school ___ |
|  |  | High School or Technical School ___ |
|  |  | Bachelor's Degree___ Advanced Degree___ |
|  |  |  |
| Monthly household income: | ______ | RMB. Per month. |

THIS QUESTIONNAIRE WILL BE MOSTLY ABOUT YOUR BREATHING CONDITION. WHEREVER POSSIBLE, PLEASE ANSWER 'YES' OR 'NO', OR DON’T KNOW.

1. Have you had wheezing or whistling in your chest at any time within NO YES

this winter? [ ] [ ]

***IF 'NO' GO TO QUESTION 2, IF 'YES', PLEASE ANSWER THE FOLLOWING QUESTIONS:***

1.1Have you been at all breathless when the wheezing noise was NO YES present within this winter? [ ] [ ]

1.2Have you had this wheezing or whistling when you did not have NO YES

a cold within this winter? [ ] [ ]

1. Have you woken up with a feeling of tightness in your chest NO YES

in the morning at any day within this winter? [ ] [ ]

1. Have you woken up with a feeling of tightness in your chest at any NO YES

time within this winter? [ ] [ ]

1. Have you been woken by an attack of coughing at any time NO YES

within this winter? [ ] [ ]

NO YES

1. Have you ever had chronic bronchitis in the last 12 months? [ ] [ ]

***IF 'NO' GO TO QUESTION 6, IF 'YES', PLEASE ANSWER THE FOLLOWING QUESTIONS:***

5.1 Was this confirmed by a doctor (including Chinese NO YES

medicine practitioners)? [ ] [ ]

NO YES

1. Have you ever had emphysema in the last 12 months? [ ] [ ]

***IF 'NO' GO TO QUESTION 7, IF 'YES', PLEASE ANSWER THE FOLLOWING QUESTIONS:***

6.1 Was this confirmed by a doctor (including Chinese NO YES

medicine practitioners)? [ ] [ ]

1. Have you ever had asthma? NO YES

[ ] [ ]

***IF 'NO' GO TO QUESTION 8, IF 'YES',*** ***PLEASE ANSWER THE FOLLOWING QUESTIONS:***

7.1 Was this confirmed by a doctor (including Chinese NO YES

medicine practitioners)? [ ] [ ]

7.2 How old were you when you had your first attack of asthma? YEARS

[ ]

7.3 Which season(s) of the year do you usually have attacks of asthma? NO YES

- - 1. Spring (March-May) [ ] [ ]
    2. Summer (June-August) [ ] [ ]
    3. Autumn (September-November) [ ] [ ]
    4. Winter (December-February) [ ] [ ]

1. Have you had an attack of asthma during this winter? NO YES

***IF 'NO' GO TO QUESTION 9, IF 'YES', PLEASE*** [ ] [ ]

***ANSWER THE FOLLOWING QUESTIONS:***

- 1. Was this confirmed by a doctor (including Chinese NO YES medicine practioners) within this winter? [ ] [ ]

8.2 Are you currently taking any medicine including inhalers? NO YES

[ ] [ ]

1. The kitchen in your house is NO YES
   - 1. separated from other rooms [ ] [ ]
     2. in the living room or bedroom [ ] [ ]
2. How large is the kitchen at your house?______________ m^2^
3. How often do you open kitchen windows while TICK ONE BOX ONLY

cooking within this winter?

- - 1. never [ ]
    2. occasionally [ ]
    3. sometimes [ ]
    4. most of the time [ ]
    5. always [ ]

1. What kind of stove do you mostly use for cooking and warming in kitchen

within this winter? TICK ONE BOX ONLY

1. coal [ ]
2. gas [ ]
3. electric [ ]
4. kerosene [ ]
5. microwave [ ]
6. How often have you spent cooking with your stove over the last winter?

TICK ONE BOX ONLY

1. cooked daily [ ]
2. cooked most of the time [ ]
3. cooked sometimes [ ]
4. cooked occasionally [ ]
5. do not cook [ ]

13.1 On average how long have you spent cooking with your stove each day over  the last winter? TICK ONE BOX ONLY

1. <30 minutes [ ]
2. 30-60 minutes [ ]
3. >60 minutes [ ]
4. When cooking, do you use the fan or range hood  TICK ONE BOX ONLY
5. all of the time? [ ]
6. some of the time? [ ]
7. few of the time? [ ]
8. none of the time? [ ]

14.1 How often did the presence of cooking oil fumes in your kitchen

cause the irritation of your respiratory tracts? TICK ONE BOX ONLY

1. frequently or sometimes [ ]
2. seldom or never [ ]

1. How often did you typically use such warming appliance over the last winter? NO YES
2. heated daily [ ] [ ]
3. heated most of the time [ ] [ ]
4. heated sometimes [ ] [ ]
5. heated occasionally [ ] [ ]
   1. Is smoke removed by a chimney or hood?  TICK ONE BOX ONLY
6. chimney [ ]
7. hood [ ]
8. both [ ]
9. neither [ ]

1. Is your kitchen haunted with pests (cockroaches or rodents) within this winter? TICK ONE BOX ONLY
   - 1. all of the time [ ]
     2. most of the time [ ]
     3. some of the time [ ]
     4. few of the time [ ]
     5. none of the time [ ]

1. How many person(s) did you share in your bedroom within this winter?  NO YES
   - 1. ≥3 persons [ ] [ ]
     2. ＜3 persons [ ] [ ]

1. Which of the following appliances do you mostly use for heating in the bedroom during the winter? TICK ONE BOX ONLY
2. coal stove, coke or wood fire [ ]
3. gas fire [ ]
4. electric heater [ ]
5. paraffin heater [ ]
6. oil-fired boiler [ ]
7. no heating appliance [ ]

18.1 How often did you typically use such heating appliance over the last winter? NO YES

- 1. heated daily [ ] [ ]
  2. heated most of the time [ ] [ ]
  3. heated sometimes [ ] [ ]
  4. heated occasionally [ ] [ ]

1. Are there fitted carpets or rug in your bedroom within this winter? NO YES [ ] [ ]

***IF 'NO' GO TO QUESTION 20, IF 'YES', PLEASE ANSWER THE FOLLOWING QUESTIONS:***

19.1How old is the oldest carpet or rug in your bedroom?

TICK ONE BOX ONLY

- - 1. less than one year [ ]
    2. 1-5 years old [ ]
    3. more than 5 years old [ ]

1. Of what material is your mattress made of within this winter? NO YES
2. foam [ ] [ ]
3. feather [ ] [ ]
4. grass/grain husks [ ] [ ]
5. pieces of cloth [ ] [ ]
6. hair piece [ ] [ ]
7. no mattress [ ] [ ]
8. How old is your mattress? TICK ONE BOX ONLY
   - 1. less than one year [ ]
     2. 1-5 years old [ ]
     3. more than 5 years old [ ]
9. What are your blankets made of within this winter TICK ONE BOX ONLY
10. cotton [ ]
11. wool [ ]
12. feather [ ]
13. no blanket [ ]
14. Is your blanket fluffy within this winter? NO YES DON’T KNOW

[ ] [ ] [ ]

1. What is your pillow stuffed with within this winter? NO YES
   - 1. foam [ ] [ ]
     2. feather [ ] [ ]
     3. grass [ ] [ ]
     4. pieces of cloth [ ] [ ]
     5. no pillow [ ] [ ]
2. Do you keep a pet (cat, dog or bird) within this winter? NO YES

[ ] [ ]

***IF 'NO' GO TO QUESTION 26, IF 'YES', PLEASE ANSWER THE FOLLOWING QUESTIONS:*** NO YES

25.1Is your pet allowed in the bedroom? [ ] [ ]

1. Has there been any damages related to water in the NO YES DON’T KNOW

building, for example, broken water pipes, leaks [ ] [ ] [ ]

or floods in the bedroom within this winter?

1. Does bedroom at your home smell musty NO YES DON’T KNOW

within this winter? [ ] [ ] [ ]

1. Has there been any mould or mildew on any surface, NO YES DON’T KNOW

other than food, inside your bedroom within this winter?[ ] [ ] [ ]

1. Have you purchased new furniture (e.g. wardrobe, table and chair) NO YES and placed them in your bedroom within this winter? [ ] [ ]
2. Is there any interior decoration and fitment in your bedroom (e.g. limewater/paint, laying wooden floors and wallpaper) within this NO YES

winter? [ ] [ ]

NO YES

1. Have you ever smoked for as long as 6-month continuous period? [ ] [ ]

***['YES' means at least 100 cigarettes in lifetime, or at least one cigarette per day]***

***IF 'NO' GO TO QUESTION 33, IF 'YES',*** ***PLEASE ANSWER THE FOLLOWING QUESTIONS:***  YEARS

31.1How old were you when you started smoking? [ ]

31.2 Do you smoke cigarettes now? NO YES

[ ] [ ]

***IF 'NO' GO TO QUESTION 32, IF 'YES',*** ***PLEASE ANSWER THE FOLLOWING QUESTIONS:***

- - 1. Have you had smoked at least 100 (5 packs) NO YES

cigarettes in your lifetime? [ ] [ ]

- - 1. Do you smoke on a daily basis within this winter? NOYES [ ] [ ]

***IF 'NO' GO TO QUESTION 32, IF 'YES', PLEASE ANSWER THE FOLLOWING QUESTIONS:***

- - - 1. How many cigarettes per day do you now NUMBER

smoke on average? [ ] [ ]

1. Have you stopped or cut down smoking? NO YES [ ] [ ]

***IF 'NO' GO TO QUESTION 33, IF 'YES', PLEASE ANSWER THE FOLLOWING QUESTIONS:***

32.1Have you had stopped smoking at least 1 year before the survey? NO YES

[ ] [ ]

32.2How old were you when you stopped or cut down smoking? YEARS [ ]

1. Have you been regularly exposed to tobacco smoke within this winter NO YES

months? ***['Regularly' means on most days or nights]***  [ ] [ ]  ***IF 'NO' GO TO QUESTION 34, IF 'YES', PLEASE ANSWER THE FOLLOWING QUESTIONS:***

33.1Have you at any point within the winter month regularly spent time in the same room with smoker/s who was/were smoking cigarettes, for more than 15 minutes (or the time for about one cigarette), at least three times per week? NO YES [ ] [ ]

1. Not counting yourself, how many people in your household NUMBER

smoke regularly within this winter? [ ][ ]

1. How many hours per day are you exposed to other people's HOURS

tobacco smoke in your household within this winter? [ ][ ]
