# Supplementary material for: Correlation between indoor air pollution and adult respiratory health in Zunyi City in Southwest China: situation in two different seasons
Source: BMC Public Health. 2019 Jun 10;19:723. doi: 10.1186/s12889-019-7063-z (PMC6558730; doi:10.1186/s12889-019-7063-z)
Supplement: Supplementary file 3 — Instructions for the questionnaire and exposure score. (DOCX 32 kb) [file 12889_2019_7063_MOESM3_ESM.docx]

Additional file 3:

**Questionnaire Instructions**

Question 1 intended to identify participants who have wheeze with breathlessness and/or wheeze in the absence of colds. No distinction was made between those who only wheeze during the day and those who only wheeze at night.

Question 2-3 referred to waking up with tightness in the chest in the morning at any day or at any time regardless of whether the person had a cold or not.

Question 4 aimed to assess waking up from sleep with cough. A cough with their first smoke or on going out of doors was included. Clearing the throat or a single cough was excluded.

Question 5-6 aimed to screen for chronic bronchitis and emphysema diagnosed by physician. Subject was excluded from this study if he/she had a positive answer to the question 5 and its subset question 5.1. Similarly, subject was also excluded when he/she had a positive answer to the question 6 and its subset question 6.1.

Question 7-8 were to screen for ever and current asthma diagnosed by physician. Asthma was defined with a positive answer to the question 8 and its subset question 8.1. Question 8.2 was asking about medication used for asthma.

Question 9 was to identify the location of kitchen in relation to bedroom. The location of kitchen associated with bedroom may have adverse effects on resident’s pulmonary health.

Question 9: If kitchen in the house was separated from other rooms, the kitchen exposure value was recorded as 0; if the kitchen was in the living room or bedroom, the exposure value was recorded as 1.

Questions 10 and 11 gathered information on kitchen dimension and ventilation. The dimension of kitchen and frequency of opening kitchen windows while cooking may be critical factors resulting in the impairment of resident’s respiratory health.

Question 10：If kitchen area in the house was less than 4 m^2^, the kitchen exposure value was recorded as 1; if the kitchen area in the house was more than or equal to 4 m^2^, the exposure value was recorded as 0. The Chinese state-level design code for civil buildings (GB 50096-1999) stipulates the size of kitchen should not be less than 4 square meter (Ministry of Construction People's Republic of China 2003).

Question 11: If the respondent always opened kitchen window while cooking, kitchen exposure value was recorded as 0; if the respondent most of the time opened kitchen window while cooking, the exposure value was recorded as 1; if the respondent sometimes opened kitchen window while cooking, the exposure value was recorded as 2; If the respondent occasionally opened kitchen window while cooking, the exposure value was recorded as 3; If the respondent had never opened kitchen window while cooking, the exposure value was recorded as 4.

Kitchen windows never open while cooking was defined as kitchen windows never open at all every day in a week; Kitchen windows open occasionally while cooking was defined as kitchen windows open 1-2 days in a week; Kitchen windows open sometimes while cooking was defined as kitchen windows open 3-4 days in a week; Kitchen windows open most of the time while cooking was defined as kitchen windows open 5-6 days in a week; Kitchen windows open always while cooking was defined as kitchen windows open all the time in a week.

Questions 12-15 dealt with the type of cooking and heating appliances, frequency and period of cooking exposure in kitchen. Coal and propane are the most popular fuel for cooking, heating and water heating in Zunyi. The burning of fuels may emit air pollutants such as NO_2_, CO, PM, which can irritate the respiratory airways.

Question 12: If the respondent mostly used coal stove for cooking, the kitchen exposure value was recorded as 2; if the respondent mostly used gas or kerosene stove for cooking, the exposure value was recorded as 1; and if the respondent mostly used electric or microwave stove for cooking, the exposure value was recorded as 0.

Question 13: If the respondent cooked daily with stove, the kitchen exposure value was recorded as 4; if the respondent cooked most of the time with stove, the exposure value was recorded as 3; if the respondent cooked sometimes with stove, the exposure value was recorded as 2; if the respondent cooked occasionally with stove, the exposure value was recorded as 1; if the respondent did not cook with stove at all, the exposure value was recorded as 0.

Cooking with stove daily was defined as cooking with stove everyday in a week; cooking with stove most of the time was defined as cooking with stove 5-6 days in a week; cooking with stove sometimes was defined as cooking with stove 3-4 days in a week; cooking with stove occasionally was defined as cooking with stove 1-2 days in a week; did not cook with stove was defined as not cooking with stove any day in a week.

Question 13.1: If on average the respondent spent less than 30 minutes cooking with stove each day, the kitchen exposure value was recorded as 0; if the respondent spent 30-60 minutes cooking with stove each day, the exposure value was recorded as 1; if the respondent spent more than 60 minutes cooking with stove each day, the exposure value was recorded as 2.

Question 14: When cooking, if the respondent used the fan or range hood all of the time, the kitchen exposure value was recorded as 0; if the respondent used the fan or range hood some of the time, the exposure value was recorded as 1; if the respondent used the fan or range hood a few times, the exposure value was recorded as 2; if the respondent used the fan or range hood none of the time, the exposure value was recorded as 3.

When cooking, using the fan or range hood all of the time was defined as the time using the fan or range hood occupies ≥80 percent of cooking time; using the fan or range hood some of the time was defined as the time using the fan or range hood occupies 20-80 percent of cooking time; using the fan or range hood a few times was defined as the time using the fan or range hood occupies ≤20 percent of cooking time; using the fan or range hood none of the time was defined as not using fan or range hood at all.

Question 14.1 If the presence of cooking oil fumes in kitchen caused the irritation of the participant’s respiratory tracts frequently or sometimes, the kitchen exposure value was recorded as 1; If the presence of cooking oil fumes in kitchen had never or seldom caused the irritation of the participant’s respiratory tracts, the kitchen exposure value was recorded as 0.

Cooking oil fumes caused the irritation of the participant’s respiratory tracts frequently was defined as cooking oil fumes caused the irritation of the participant’s respiratory tracts 5-7 days in a week; cooking oil fumes caused the irritation of the participant’s respiratory tracts sometimes was defined as cooking oil fumes caused the irritation of the participant’s respiratory tracts 3-4 days in a week; cooking oil fumes caused the irritation of the participant’s respiratory tracts seldom was defined as cooking oil fumes caused the irritation of the participant’s respiratory tracts 1-2 days in a week; cooking oil fumes had never caused the irritation of the participant’s respiratory tracts seldom was defined as cooking oil fumes did not cause the irritation of the participant’s respiratory tracts any day in a week.

Question 15: If the respondent typically used such heating appliance daily, the kitchen exposure value was recorded as 3; if the respondent typically used such heating appliance most of the time, the exposure value was recorded as 2; if the respondent typically used such heating appliance sometimes, the exposure value was recorded as 1; if the respondent typically used such heating appliance occasionally, the exposure value was recorded as 0.

Typically, using such heating appliance daily was defined as using such heating appliance everyday in a week; using such heating appliance most of time was defined as using the heating appliance 5-6 days in a week; using such heating appliance sometimes was defined as using the heating appliance 3-4 days in a week; using such heating appliance occasionally was defined as using the heating appliance 1-2 days in a week.

Question 15.1: If smoke was removed by both chimney and hood, the exposure value was recorded as 0; if smoke was removed by a chimney or hood, the kitchen exposure value was recorded as 1; if smoke was not removed by either chimney or hood (neither), the exposure value was recorded as 2.

Question 16 (Question 15 in summer questionnaire) dealt with pests (cockroaches or rodents) haunted in kitchen. Droppings or body parts of pests can become asthma triggers. Cockroaches are likely to contribute to asthma problems in inner city area.

Question 16: If the kitchen was haunted with pests all of the time, the kitchen exposure value was recorded as 4; if the kitchen was haunted with pests most of the time, the exposure value was recorded as 3; if the kitchen was haunted with pests some of the time, the exposure value was recorded as 2; if the kitchen was haunted with pests few of the time, the exposure value was recorded as 1; if the kitchen was haunted with pests none of the time, the exposure value was recorded as 0.

The kitchen was haunted with pests all of the time was defined as the kitchen was haunted with pests everyday in a week; the kitchen was haunted with pests most of the time was defined as the kitchen is haunted with pests 5-6 days in a week; the kitchen was haunted with pests some of the time is defined as the kitchen was haunted with pests 3-4 days in a week; the kitchen was haunted with pests few of the time was defined as the kitchen was haunted with pests 1-2 days in a week; the kitchen was haunted with pests none of the time was defined as the kitchen was not haunted with pests at all in a week.

Question 17 (Question 16 in summer questionnaire) was related to number of people living in the same bedroom (crowding). Crowding in bedroom might be related to high risk of asthma morbidity.

Question 17: If the respondent’s bedroom was shared by more than and equal to 3 persons, the bedroom exposure value was recorded as 1; if the respondent’s bedroom was shared by less than 3 persons, the bedroom exposure value was recorded as 0.

Question 18 was related to type of heating appliances, and frequency of heating exposure in bedroom. The use of coal or propane stove in bedroom could increase high concentration of air pollutants, particularly in winter month.

Question 18: If the respondent used coal stove, coke or wood fire for heating in the bedroom, the bedroom exposure value was recorded as 2; if the respondent used gas fire, paraffin heater or oil-fired boiler for heating, the exposure value was recorded as 1; if the respondent used electric heater for heating or no heating appliance at all, the exposure value was recorded as 0.

Question 18.1: If the respondent used heating appliance daily, the bedroom exposure value was recorded as 3; if the respondent used heating appliance most of time, the exposure value was recorded as 2; if the respondent used heating appliance sometimes, the exposure value was recorded as 1; if the respondent used heating appliance occasionally, the exposure value was recorded as 0.

Using heating appliance in bedroom daily was defined as using the heating appliance everyday in a week; using heating appliance in bedroom most of time was defined as using the heating appliance 5-6 days in a week; using heating appliance in bedroom sometimes was defined as using the heating appliance 3-4 days in a week; using the heating appliance in bedroom occasionally was defined as using the heating appliance 1-2 days in a week.

Questions 19-25 (Questions 17-23 in summer questionnaire) were to identify the use of floor carpets, mattress, fluffy blankets, pillow, and the exposure to furry pets. These items are the ideal places for dust, insect wastes, and insects, such as house dust mites, and all known allergens. Exposure to these particulates can trigger asthma and asthma-related symptoms.

Question 19 (Question 18 in summer questionnaire): If the respondent bedroom had fitted carpets, the bedroom exposure value was recorded as 1; if the respondent bedroom did not have fitted carpets, the exposure value was recorded as 0.

Question 19.1 (Question 17.1 in summer questionnaire): If the oldest carpet or rug in respondent bedroom was less than one year, the bedroom exposure value was recorded as 0; if the oldest carpet or rug in respondent bedroom was 1-5 years old, the exposure value was recorded as 1; if the oldest carpet or rug in respondent bedroom was more than 5 years old, the exposure value was recorded as 2.

Question 20 (Question 18 in summer questionnaire): If the respondent mattress was made of feather or hair piece, the bedroom exposure value was recorded as 2; if the respondent mattress was made of foam or grass/grain husks, the exposure value was recorded as 1; if the respondent mattress was made of pieces of cloth or no mattress, the exposure value was recorded as 0.

Question 21 (Question 19 in summer questionnaire): If the respondent mattress was more than 5 years old, the bedroom exposure value was recorded as 2; if the respondent mattress was 1-5 years old, the exposure value was recorded as 1; if the respondent mattress was less than one year old, the exposure value was recorded as 0.

Question 22 (Question 20 in summer questionnaire): If the respondent blankets were made of feathers or wool, the bedroom exposure value was recorded as 1; if the respondent blankets were made of cotton or no blanket, the exposure value was recorded as 0.

Question 23 (Question 21 in summer questionnaire): If the respondent blanket was fluffy, the exposure value was recorded as 1; if the respondent blanket was not fluffy or the respondent does not know whether blanket was fluffy, the exposure value was recorded as 0.

Question 24 (Question 22 in summer questionnaire): If the respondent pillow was stuffed with feather, the bedroom exposure value was recorded as 2; if the respondent pillow was stuffed with grass or foam, the exposure value was recorded as 1; if the respondent pillow was stuffed with pieces of cloth or no pillow at all, the exposure value was recorded as 0.

Question 25 (Question 23 in summer questionnaire): If the respondent kept a pet (cat, dog or bird) and the pet was allowed in the bedroom, the bedroom exposure value was recorded as 1; if the respondent did not kept a pet, the exposure value was recorded as 0.

Questions 26-28 (Questions 24-26 in summer questionnaire) gather information on the degree of bedroom dampness (self-reported dampness, appearance of mold, musty smell and spots of water damage). Dampness has been shown to be related to asthma and asthma-related symptoms.

Question 26 (Question 24 in summer questionnaire): If there had been any water damage to the building in the bedroom, the bedroom exposure value was recorded as 1; if there had not been any water damage to the building in the bedroom, or the respondent did not know whether there had been any water damage to the building in the bedroom, the exposure value was recorded as 0.

Question 27 (Question 25 in summer questionnaire): If the respondent bedroom at home smelt musty, the bedroom exposure value was recorded as 1; if the respondent bedroom at home did not smell musty, or the respondent did not know whether the bedroom smell musty or not, the exposure value was recorded as 0.

Question 28 (Question 26 in summer questionnaire): If there had been any mould or mildew on any surfaces, other than food, inside the respondent bedroom, the bedroom exposure value was recorded as 1; if there had not been any mould or mildew on any surfaces inside the respondent bedroom, or the respondent did not know whether there had been any mould or mildew on any surfaces, the exposure value was recorded as 0.

Questions 29-30 (Questions 27-28 in summer questionnaire) dealt with purchase of new furniture, interior decoration and fitment in bedroom. New furniture, decoration and fitment material such as plywood may emit high concentration of VOCs such as benzene and toluene in house. VOCs is a well-known airborne contaminant irritating the airways and triggering respiratory symptoms.

Question 29 (Question 27 in summer questionnaire): If the respondent had purchased new furniture (e.g. wardrobe, table and chair) and placed in the bedroom, the bedroom exposure value was recorded as 1; if the respondent had not purchased new furniture in the bedroom, the exposure value was recorded as 0.

Question 30 (Question 28 in summer questionnaire): If there was any interior decoration and fitment in the respondent bedroom (e.g. limewater/paint, laying wooden floors and wallpaper), the bedroom exposure value was recorded as 1; if there was no interior decoration and fitment in the respondent bedroom, the exposure value was recorded as 0.

Question 29 (only in summer questionnaire) was related to the use of mosquito repellent.

Question 29: If the respondent used mosquitoes killing spray or coil incense to keep away from mosquito biting, the bedroom exposure value was recorded as 1; if the respondent used mosquito net or no method to keep away from mosquito biting, the bedroom exposure value was recorded as 0.

Questions 31-35 (Questions 30-34 in summer questionnaire) were to gather information on the ETS exposure (passive smoking and active smoking). Exposure to environmental tobacco smoking had been found to be associated with respiratory symptoms.
